# Ultrasound-assisted carbon ion dosimetry and range measurement using injectable polymer-shelled phase-change nanodroplets: in vitro study

**DOI:** 10.1038/s41598-022-11524-x

**Published:** 2022-05-14

**Authors:** Yosra Toumia, Marco Pullia, Fabio Domenici, Angelica Facoetti, Michele Ferrarini, Sophie V. Heymans, Bram Carlier, Koen Van Den Abeele, Edmond Sterpin, Jan D’hooge, Emiliano D’Agostino, Gaio Paradossi

**Affiliations:** 1 Department of Chemical Science and Technologies, University of Rome Tor Vergata, 00133 Rome, Italy; 2 National Institute for Nuclear Physics, INFN Sez. Roma Tor Vergata, 00133 Rome, Italy; 3 Fondazione CNAO, The National Center of Oncological Hadrontherapy, 27100 Pavia, Italy; 4 Department of Physics, KU Leuven Campus Kulak, Kortrijk, Belgium; 5 Department of Cardiovascular Sciences, KU Leuven, Leuven, Belgium; 6 Biomedical Engineering, Department of Cardiology, Erasmus MC University Medical Center, Rotterdam, The Netherlands; 7 Department of Oncology, KU Leuven, Leuven, Belgium; 8 DoseVue, Hasselt, Belgium

**Keywords:** Medical research, Oncology, Chemistry, Materials science, Physics

## Abstract

Methods allowing for in situ dosimetry and range verification are essential in radiotherapy to reduce the safety margins required to account for uncertainties introduced in the entire treatment workflow. This study suggests a non-invasive dosimetry concept for carbon ion radiotherapy based on phase-change ultrasound contrast agents. Injectable nanodroplets made of a metastable perfluorobutane (PFB) liquid core, stabilized with a crosslinked poly(vinylalcohol) shell, are vaporized at physiological temperature when exposed to carbon ion radiation (C-ions), converting them into echogenic microbubbles. Nanodroplets, embedded in tissue-mimicking phantoms, are exposed at 37 °C to a 312 MeV/u clinical C-ions beam at different doses between 0.1 and 4 Gy. The evaluation of the contrast enhancement from ultrasound imaging of the phantoms, pre- and post-irradiation, reveals a significant radiation-triggered nanodroplets vaporization occurring at the C-ions Bragg peak with sub-millimeter shift reproducibility and dose dependency. The specific response of the nanodroplets to C-ions is further confirmed by varying the phantom position, the beam range, and by performing spread-out Bragg peak irradiation. The nanodroplets’ response to C-ions is influenced by their concentration and is dose rate independent. These early findings show the ground-breaking potential of polymer-shelled PFB nanodroplets to enable in vivo carbon ion dosimetry and range verification.

## Introduction

Advanced radiotherapy using heavy charged particle beams (i.e. hadron therapy) such as protons and carbon ions (C-ions) has become recently clinically accessible and is growing worldwide in a continuous effort aiming to increase the number of therapeutic options for tumors that are resistant to the traditional treatments^1,2^. Besides, hadron therapy is considered to be more beneficial in treating cancers that are nearby critical organs (e.g. left breast cancer bordering the heart) compared to conventional radiotherapy. Unlikely X-ray photons, charged particles diffuse less when penetrating the tissues and deposit the maximum energy in a few millimeters-wide interval just before stopping, thereby releasing the majority of their energy in a highly localized sharp distal dose fall-off known as the Bragg peak^3–5^. As a result, the dose distributions achieved with hadron beams are superior to those achievable by photon beams due to their finite and narrow deposition range (i.e. limited lateral spread) in the body. Although both C-ions and protons have similar physical advantages compared to X-rays, the radiobiological properties of C-ions are quite distinct from protons, and are considered as an innovation for the treatment of radio-resistant cancers typically associated with negative prognosis and high mortality. The use of C-ions in radiotherapy was first suggested by Cornelius A. Tobias, who postulated that heavier ions could be more efficient than protons^6^. The major differences in dose-distribution between these two types of radiation reside in the fact that C-ions feature a small fragmentation tail beyond the distal fall-off. Besides, in the lateral direction, C-ions are characterized by a steeper fall-off than a proton beam, and have superior conformality to the target due to a notably narrower Bragg peak, which makes them able to more effectively hit the tumor mass and better spare the healthy tissues in front and behind the tumor^7^. Moreover, the linear energy transfer (LET), i.e. the density of energy deposited by the charged particle in the traversed material per unit length, produced by primary protons is low compared to the high LET C-ions^7–9^. C-ions induce the maximal relative biological effectiveness (RBE) at the Bragg peak, and demonstrate an optimal efficacy against resistant tumors when the LET value is about 150–200 keV/μm^10,11^. Furthermore, recent research advances showed that the radiobiological properties of densely ionizing carbon can lead to additional therapeutic effects in cancer therapy, enhancing the immune response and reducing the angiogenesis and metastatic potential^7^. The interest gained by the clinical potential of C-ions is reflected in the increasing numbers of patients treated in the last two decades. Phase I and II clinical trials in Japan have shown promising results for patients with localized advanced pancreatic cancer. Other phase II clinical trials were recently conducted in Germany to confirm these findings^12^. However, according to the Particle Therapy Co-Operative Group (PTCOG) the number of currently active centers is still limited to 12 facilities across the world; mainly in Europe (Italy, Germany, Austria) and Asia (China and Japan), while centers are under development in the USA and in France^13^.

As of today, one of the most crucial challenges of all particle therapy plans, including C-ion radiation therapy, remains the patient *in-vivo* dosimetry and range verification. In practice, range uncertainties in treatment delivery, e.g. deriving from errors in the setup or from anatomical motions of the patient (i.e. organ/target shifting), restrict the full benefits of C-ion radiation therapy^14^. Thus, the treatment efficacy could still be further enhanced if the safety margins could be reduced, and if errors in the dose distribution could be detected and compensated in real-time to avoid undesired exposure^15–17^. Often, Monte Carlo simulations or absolute range measurement of the particle beam allow for an accurate treatment plan. Nonetheless, this approach is typically only applicable offline and remains theoretical^18,19^. Prompt gamma imaging and positron emission tomography (PET) have been proposed as alternative verification techniques in hadron therapy, although till now they are not yet routinely adopted in clinics^20,21^. Recently, Sun et al. reported on the feasibility of using PET/CT images shortly after carbon-ion radiotherapy for in vivo 3-D dose verification, however, the conclusion was that further studies are needed to be able to correlate the positron distribution to the C-ions dose distribution^20^. Currently, a clinical trial is ongoing at the Oncological Hadron Therapy Center (CNAO, IT) employing a PET detector in combination with a charged particle tracer (*ClinicalTrials.gov ID:NCT03662373).*

In recent publications, we have successfully demonstrated the use of superheated perfluorocarbon (PFC) nanodroplets (i.e. with PFCs being in the liquid phase above their boiling point), with an average size of a few hundred nanometers^22–24^, as a novel concept for radiation dosimetry assisted by ultrasound imaging^25–27^. In particular, perfluorobutane (C_4_F_10_) nanodroplets (PFB NDs, b.p. − 2 °C) encapsulated by a shell of poly(vinylalcohol) (PVA) proved promising and yielded reproducible results for range verification in a passively scattered proton beam. Prior to our conducted studies, similar type NDs, generally consisting of a PFC liquid core stabilized with lipid or polymer shell, had been introduced by several researchers as the 'next generation' of ultrasound (US) contrast agents^28,29^; whereby the liquid to gas phase transition could be triggered acoustically or optically to generate microbubbles providing US contrast^30,31^. As an extension, we have proven, using proton and photon beam irradiation, that the vaporization of metastable NDs could also be triggered by ionizing radiation in line with a similar mechanism of superheated drop detectors where vaporization occurs when the radiation’ LET is sufficiently high to induce homogenous nucleation of vapor embryos in the superheated core^25,32–34^. Accordingly, the key parameters driving the radiation-induced phase-change are the LET of the individual charged particles present in the radiation beam and the degree of superheat (*s*) of the droplet liquid core, defined by Eq. (1) ^33^.

1 $$s = \frac{{T - T_{b} }}{{T_{c} - T_{b} }}$$

where *T*_b_ is the boiling temperature of the considered liquid, *T*_c_ is the critical temperature and T is the experimental temperature during radiation exposure.

The aforementioned PVA shelled PFB NDs (PVA/PFB) were shown to be sensitive to primary protons when increasing the temperature up to 50 °C, while at physiological temperature vaporization was only triggered by secondary produced high LET nuclear recoils. In both cases, the vaporization response of the NDs along the beam path, detected by ultrasonography, exhibited a highly reproducible (< 1 mm) relationship between the actual proton range and the generated US contrast profiles^26^. In the current paper, we extend the previous studies to investigate the sensitivity of PVA/PFB NDs to a clinical C-ion source by means of US imaging. Due to the high track-averaged LET achieved by C-ions, we expect the NDs to be directly sensitive to primary C-ions at physiological temperature, without modulation of their degree of superheat, and vaporization events to occur primarily at the Bragg peak where these particles reach their maximum LET, rather than possibly by only secondary products/recoils as in the case of protons. To this aim, NDs, homogenously dispersed in tissue-mimicking phantoms of poly(acrylamide) hydrogel (PAM), were irradiated at body temperature using various clinically-relevant doses. Subsequently, the resulting US contrast from the NDs vaporization was correlated to the C-ions dose and to the predicted range. We applied both pristine and spread-out Bragg peaks irradiations, and modified the C-ions range as well as the phantom position within the beam to confirm the specific response of the PVA/PFB NDs. Finally, we examined the effect of concentration and dose rate on the performance of the NDs dosimeter to C-ions. To the best of our knowledge, this work presents the first study describing the effect of C-ions beam on phase-change US contrast agents for dosimetry purposes.

## Methods

### PVA/PFB nanodroplets preparation and quantification

The PVA shelled PFB NDs were prepared as described elsewhere^26,27^. Typically, an aqueous solution of fully hydrolyzed PVA (Mn = 30 ± 5 kg/mol; Merck, Milan, Italy) at a concentration of 2% (w/v) is first prepared by dissolving the polymer at 80 °C. Then, NaIO_4_ (Merck, Milan, Italy) oxidant is added at a ratio of 2% (mol: mol) with respect to the PVA repeating unit. The solution is kept under stirring for 1 h to split the head-to-head sequences leading to telechelic PVA with aldehyde groups as terminals. PFB (b.p. − 2 °C, Apollo Scientific, Manchester, UK) is condensed at low temperature by fluxing it for only a few seconds in an empty glass vial sealed with a rubber septum and immersed in liquid nitrogen. Subsequently, 5 ml of the telechelic PVA solution is injected into the vial while still immersed in the liquid nitrogen, followed by an immediate sonication of the mixture for 15 min at 100% power using an ice-cold ultrasonic bath cleaner (200 W, 40 kHz, Ceia CP104, Florence, Italy). During the sonication process, the liquefied PFB is encapsulated by the PVA chains and a crosslinking acetalization occurs between the aldehyde and hydroxyl groups allowing the formation of a resistant shell. The nanodroplets are left at 4 °C for another hour to statically continue the crosslinking process prior to their washing. Finally, the PVA/PFB nanodroplets are washed with milli-Q water (18.2 MΩ cm, Pure Lab from USF, Rome, Italy) by centrifugation (5000 rpm/4080 rcf, 5 min) and stored at 4 °C for further use.

The concentration of the NDs, expressed as a numerical density (NDs/ml), was estimated by bright field/confocal fluorescence microscopy using a Neubaur counting chamber (0.1 mm × 0.0025 mm^2^), and by operating an inverted Eclipse model Ti-E microscope (Nikon Instruments, Japan), equipped with a long distance objective (S plan Fluor ELWD 40 × Ph2 ADM). The lookup table (LUT) was adjusted during image acquisition to distinguish tiny NDs spots. Finally, the average NDs count per ml was calculated on five ROIs using ImageJ freeware by adopting the pixel maxima function (see Fig. S1 in the Supplementary Information). The resulting concentration could be underestimated as the small population of NDs will be limited by the microscope resolution, i.e. 450 nm.

In addition, dynamic light scattering (Brookhaven Instruments Co. NY) was used to measure the intensity weighted size distribution of the PVA/PFB NDs suspension over 3 measurements of 3 different batches.

### Tissue-mimicking phantoms preparation

Tissue-mimicking phantoms of poly(acrylamide) hydrogels incorporating PVA/PFB NDs were prepared in rectangular cuboid poly(methyl methacrylate) (PMMA) containers, described in Fig. S2 of the Supplementary Information (inner dimensions: length = 54 mm, width = 26 mm, depth = 31 mm). All reagents were purchased from Merck (Milan, Italy). Typically, 39 ml of acrylamide solution (5% w/v) containing *N*,*N*′-methylenebisacrylamide (BIS) at a molar ratio of 1/29 (mol: mol) are degassed and filled into each container. Then, in the following order, 1 ml of ammonium persulfate (8.5% w/v), the desired volume (i.e. 10–50 μl) of PVA/PFB NDs (bulk concentration of (7 ± 0.6) × 10^9^ ND/ml), and 50 μl of tetramethylethylenediamine (TEMED) are added to trigger the radical polymerization. The mixture is gently stirred using a spatula, and is then left for 20 min at room temperature until gelation. Finally, the phantoms containing the NDs are thermalized at 37 °C in thermostatic bath (GBath 1800, MI, IT) prior to radiation exposure.

For each tested parameter, independent phantom replicas were prepared. The concentration of the NDs in the phantom was optimized to 4 × 10^6^ ND/ml for dosimetry and range verification studies to avoid a saturation of the US signal resulting in acoustic shadowing, and to enhance the signal-to-noise ratio between the echographic contrast of the vaporized NDs at the Bragg peak (signal) and the initial background of the phantom (noise).

### Carbon ions radiation exposure

**Figure 1:**
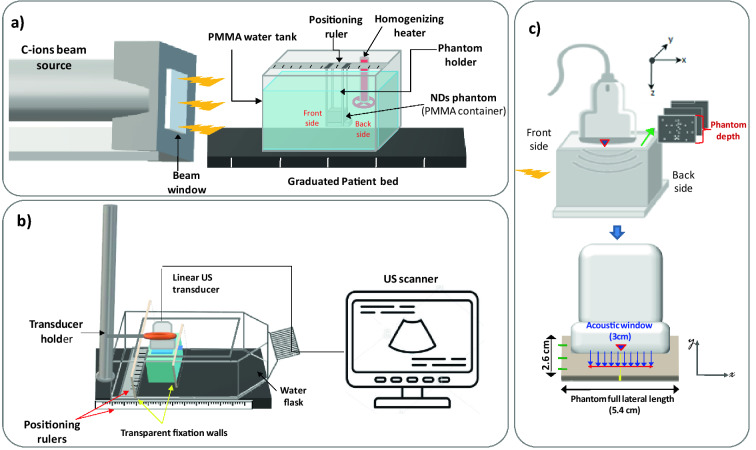
C-ions exposure experiment of PVA/PFB NDs entrapped in PAM tissue-mimicking phantoms and filled in PMMA containers: (**a**) Representative scheme of the C-ions irradiation setup; (**b**) Offline US imaging setup of PVA/PFB NDs phantoms pre- and post-irradiation at 7.5 MHz frequency and mechanical index MI = 0.1 (the probe is positioned on top of the phantom); (**c**) Depiction of the imaging scan of the phantoms along the Y axis of the PMMA container (green arrow), and of the acoustic window considered for each image (the red line indicates extent of the lateral length of the acoustic window, i.e. 3 cm). The center of the probe was aligned with the middle of the internal length of the phantom container (red triangle and yellow mark), i.e. parallel to the beam direction. The green marks indicate the probe positioning for a phantom scan through the y axis (acquisition of 3 ROIs).

Irradiation studies were carried out at the National Center for Oncological Hadron Therapy (CNAO, Pavia, Italy) using a clinical accelerator source producing a C-ions beam at an energy of 312 MeV/u. The field size was obtained by beam scanning set to 6 × 6 cm for all experiments. The experimental setup is depicted in Fig. 1: each phantom was fixed in position using a holder immersed in a PMMA water tank (model 41023 from PTW; dimensions 30 × 30 × 30 cm), equipped with a heating homogenizer (accuracy: ± 1 °C) and thermalized at 37 °C. The phantom was positioned being at the isocenter of the beam in such a way that the Bragg peak of the C-ion beam was located inside the phantom (i.e. at 144 mm and at 20 mm from the beam source, respectively for a 180 mm and 50 mm C-ions range). All phantoms were irradiated with a two-ripple filter from the thinner wall of the container, named as the front side (see Figs. 1a, and S2). The dosimetric response of the PVA/PFB NDs was assessed for different doses (reported at the Bragg peak) ranging from 0.1 to 4 Gy (i.e. corresponding to a fluence between 1.25 × 10^6^ and 5 × 10^7^ C-ions/cm^2^). The range verification potential of the NDs response to C-ions was further evaluated at a fixed single dose (i.e. 1 Gy) by (1) modifying the phantom position at the same beam range from 144 to 154 mm, (2) by performing a spread-out Bragg peak irradiation (SOBP), i.e. accumulating pristine peaks ranging from 160 to 180 mm (SOBP thickness = 10 mm), and (3) by modifying the C-ions range to 50 mm (the corresponding beam energy is 150.7 MeV/u). The parameters used for the NDs radiation sensitivity evaluation are summarized in Table 1.

**Table 1:** Experimental parameters for the evaluation of C-ions triggered PVA/PFB NDs vaporization at 37 °C and 312/u MeV beam energy.

Experiment	Dose (Gy)	Fluence (C-ions/cm^2^)	NDs concentration (× 10^6^ ND/ml)	Dose rate (%)	Beam range (mm)	Phantom entrance position (mm)	Number of irradiated phantoms
Dose effect	0.1–4	1.25 × 10^6 ^− 5 × 10^7^	**4**	**100**	**180**	**144**	8
Concentration effect	**1**	**1.25** **×** **10**^**7**^	0.8–8	100	180	144	8
Dose rate effect	1	1.25 × 10^7^	4	50	180	144	2
**Specificity and range verification**							
Phantom shift	4	5 × 10^7^	8	100	180	154	2
SOBP	1	1.25 × 10^7^	4	100	180	144	2
Range change	1	1.25 × 10^7^	4	100	50	20	2

The values highlighted in bold are considered to be the reference parameters for the different tests.

Negative control experiments were performed on non-irradiated nanodroplets phantoms that underwent the same incubation conditions in the beam room, however without exposure, as well as on a pure PAM phantom prepared without NDs which was exposed to the highest tested dose, i.e. 4 Gy.

### Carbon ions absolute range measurement

The absolute range of the carbon ions was previously calibrated during the commissioning of the CNAO center using a standard water column mod (Mirandola et al.^35^ “CNAO commissioning”). The depth energy of the Bragg peaks for the measurements described herein was determined using PeakFinder software from PTW Dosimetry Company (Freiburg, Germany).

### Data acquisition by ultrasound imaging of the phantoms

Offline ultrasound imaging of the phantoms was performed just before and immediately after C-ion irradiation (or after incubation at 37 °C for the control phantoms) by means of a clinical ultrasound scanner (Mindray DP50, China). The system was equipped with a linear array transducer (75L38EA, center frequency = 7.5 MHz) fixed on a holding stage to scan the phantoms parallel to the path traversed by the C-ions beam. For the scanning, each phantom was placed in a water flask incorporating parallel fixation walls that fit the external dimension of the PMMA container. The position of the flask was adjusted using positioning rulers to make the scan measurements repeatable (see Fig. 1b). All images were acquired using the same US gain settings. A low mechanical index value (MI = 0.1) was applied during the scans to prevent acoustic droplet vaporization that typically occurs at an MI threshold of 0.4. The ultrasound probe (imaging window of 30 mm length × 23 mm depth) was centered with respect to the middle of the internal lateral length of the phantom container and the scanning consisted in recording three parallel frames of the phantom across its width (See Fig. 1c).

### Image processing

All images acquired with the US imaging system for each phantom were analyzed using ImageJ freeware (Schindelin, 2012). The US contrast relative to microbubbles density, either derived from minor spontaneous vaporization as background noise (i.e. in the pre-radiation images and control phantoms) or generated upon C-ion exposure around the Bragg peak, was assessed by using ImageJ’ s default threshold profile function. The results are presented as grayscale value profiles (i.e. the measure of pixel brightness) extracted across the full depth as a function of the lateral length of the acoustic window for each frame of the phantom. A noise correction for each post-radiation/or incubation (i.e. for control phantoms) image was applied by subtracting the pre-irradiation/ or pre-incubation background. Subsequently, the obtained profiles from two irradiated phantoms per each dose/test and from the control phantoms were further averaged (3 frames/phantom; n = 6). The middle-label line of the phantom container matching the probe center during the imaging (see description in the caption of Fig. 1c) was used to calibrate the US scans and to convert the image coordinates of the acoustic window into the actual position within the beam path. The container wall thickness at the entrance of the beam (‘front’ side) was taken into consideration to correlate with the exact vaporization peak position. The absolute C-ion range was then compared to the extracted grey value profile of the NDs US scan. The 50% distal drop in grayscale values was calculated by taking the midpoint between the highest and lowest grey values, and used to quantify the range shift of the vaporization profile with respect to the Bragg peak. The shifts in the position of the signals were presented as mean ± standard deviation.

## Results

### Carbon ion radiation response of nanodroplets and dose dependence

**Figure 2:**
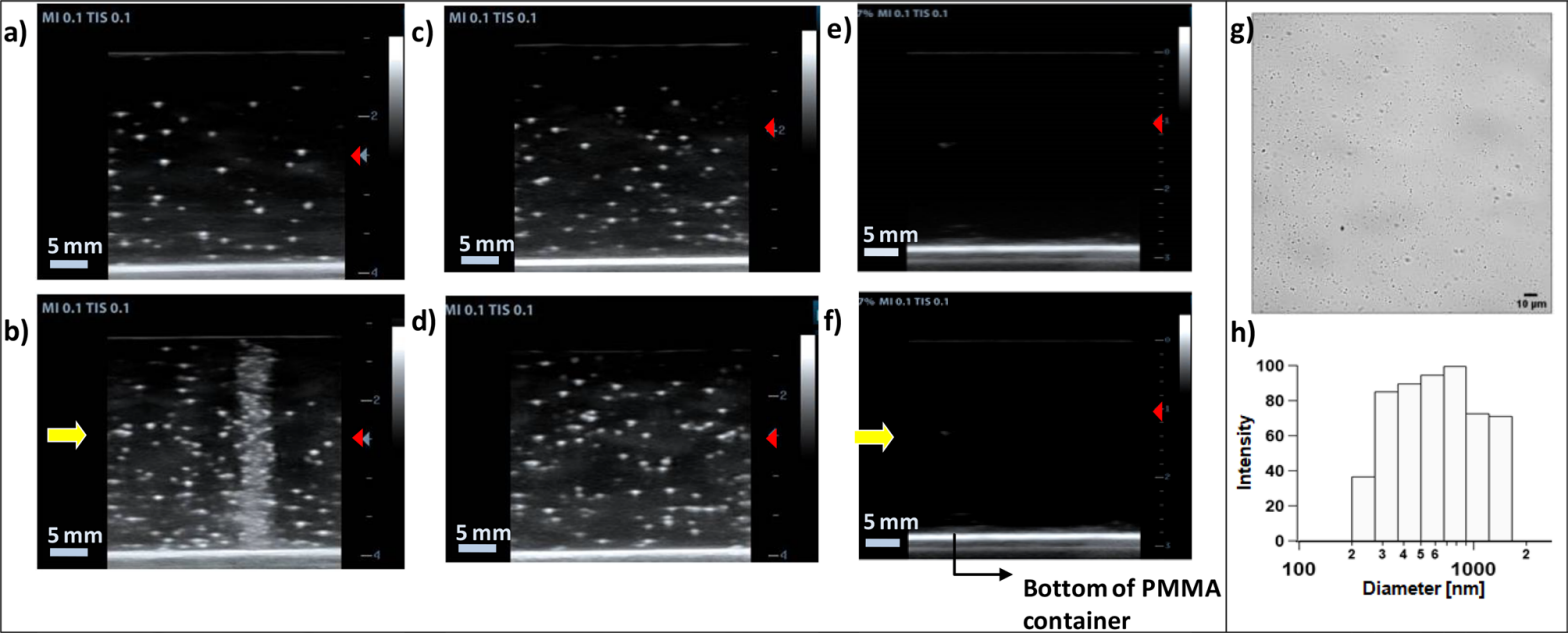
Ultrasound images of PVA/PFB NDs dispersed in PAM phantom (4 × 10^6^ ND/ml) before (**a**) and after (**b**) exposure to 312 MeV/u C-ions (4 Gy dose, 180 mm range). Corresponding images for a control phantom with dispersed PVA/PFB NDs before (**c**) and after (**d**) incubation at 37 °C (i.e. without irradiation). US images of control phantom made of pure PAM without NDs before (**e**) and after (**f**) at identical irradiation conditions. The images are acquired at 7.5 MHz with MI = 0.1. The yellow arrows indicate the beam entrance side, The red triangles indicate the US focal depth. (**g**) Oil immersion optical microscopy image of PVA/PFB NDs (objective 60 ×). (**h**) Size distribution of the nanodroplets by intensity-weighted dynamic light scattering.

Examples of US images obtained for PAM tissue-mimicking phantoms with and without PVA/PFB NDs are presented in Fig. 2. Upon thermalization @37 °C and before irradiation, both phantoms containing homogeneously dispersed NDs (optimized concentration of 4 × 10^6^ ND/ml) exhibited a similar US signal background, where only a few discernible microbubbles resulting from minor spontaneous vaporization which appear as bright spots (see Fig. 2a,c).. Prior to their phase-transition, NDs (average diameter = 700 ± 100 nm, see Fig. 2g,h) are undetectable by US. However, when converted into microbubbles after activation they provide a bright contrast due to the large acoustic impedance mismatch between the surrounding tissue-mimicking matrix and the gaseous core. The background signal from spontaneous vaporization could be decreased by size exclusion of NDs, keeping only the smallest portion which is highly stabilized by the Laplace pressure. It is worth mentioning that the appearance of speckles can be attributed to multiple microbubbles detected in the same spot, which due to their micron size cannot be resolved by the US imaging system^36^. After exposure to 4 Gy C-ions, the phantoms displayed a confined zone of high bubbles density arising at the expected end of the beam range (see Fig. 2b). On the contrary, the NDs control phantoms, after an equal incubation time at 37 °C but without radiation exposure, showed only a very slight increase in the US contrast similar to the distal region within the irradiated phantoms, i.e. beyond the Bragg peak, (see Fig. 2d). Moreover, Figs. 2e,f clearly show that no bubbles could be detected in a control PAM phantom without NDs even after exposure to 4 Gy C-ions. These results suggest, for the first time, that the vaporization of PVA/PFB NDs upon C-ions exposure originates exclusively from the interaction of the NDs with the primary charged particles depositing their maximal energy, hence triggering the nucleation of the core.

**Figure 3:**
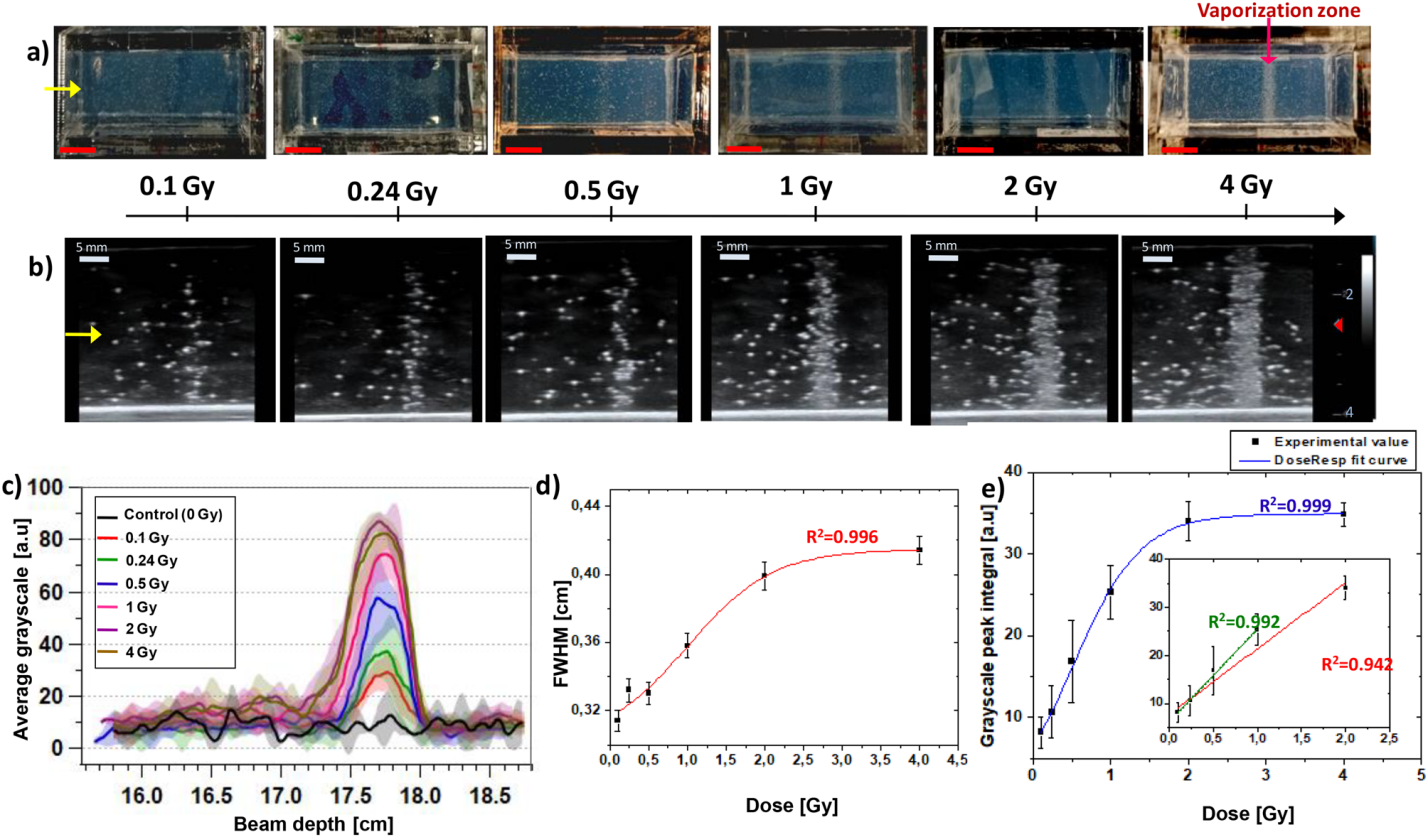
Dose effect of C-ions (312 MeV/u, 180 mm range) on the triggered vaporization of PVA/PFB NDs @ 37 °C: (**a**) Top-view photographs of independent phantoms of NDs dispersed in PAM (4 × 10^6^ NDs/ml) post-irradiation at doses between 0.1 and 4 Gy (the red scale bars correspond to 10 mm); (**b**) corresponding depth-resolved US images (7.5 MHz, MI = 0.1) of the NDs phantoms at each dose, the yellow arrow indicates the C-ions beam’ entrance side (scale bars are 5 mm); (**c**) Comparison of the average grayscale value profiles of the US images at different doses as a function of the distance traveled by C-ions (beam depth), the shaded areas correspond to the standard deviation (n = 6). (**d**) Variation of the FWHM of grayscale peaks as a function of the received C-ions dose (the red line is a dose–response fit function). (**e**) Evaluation of the peaks integrals from the average grayscale profiles as a function of C-ions dose. The inset shows the linear regression fits in the intervals of 0.1–1 Gy (green) and 0.1–2 Gy (red).

Figures 3 and S3 in the Supplementary Information illustrate the influence of the C-ions dose on the induced vaporization intensity of PVA/PFB NDs around the position of the Bragg peak. These results can be used to quantify the vaporization dose dependency limit. The dose effect on the NDs response at physiological temperature can easily be observed visually and was confirmed by US imaging of the irradiated phantoms with the same NDs concentration (i.e. 4 × 10^6^ NDs/ml). The photographs in Fig. 3a, of the top surface of the phantoms after exposure at different doses, illustrate that the evolution of the observed vaporization zone, is gradually becoming more definite and visible with the increase of the dose from 0.1 to 4 Gy. The corresponding depth-resolved US images, as illustrated in Fig. 3b, follow the same trend and reveal a gradual contrast enhancement up to a saturation at the highest dose (i.e. 4 Gy). It is worth noting that at the lowest doses, the activation of NDs into microbubbles results in visibly a narrower contrast signal at the Bragg peak. This was also confirmed by analyzing and plotting the lateral grayscale value profiles derived from the US images (see Methods/Image processing), where the observed peak in each profile is attributed to the contrast generated by the radiation-induced phase-change of the NDs. As shown in Figs. 3c,d, S4 and Tables S1, S2 of the Supplementary Information, the intensity and width of the vaporization profile peaks show a strong dependence on the exposure dose. In Fig. 3e, we evaluated the linearity of the NDs’ response by integrating the vaporization peaks as a function of the C-ions dose. The integrated profile of the generated grayscale contrast signal followed a perfect dose–response curve trend (R^2^ = 0.999, see Eq. S1in the Supplementary Information). The contrast enhancement reaches a plateau at 2 Gy dose due to the high density of generated microbubbles, causing a saturation of the acoustic signal. A very good linear behavior of the NDs’ response as a function of the dose is confirmed by the excellent linear regression fit between 0 and 1 Gy (R^2^ = 0.992). However, by expanding this range to 2 Gy, the linearity seems to be slightly bend off although the integrated US signal levels could still be considered within an acceptable confidence interval (R^2^ = 0.942).

### Influence of the nanodroplets concentration and dose rate

**Figure 4:**
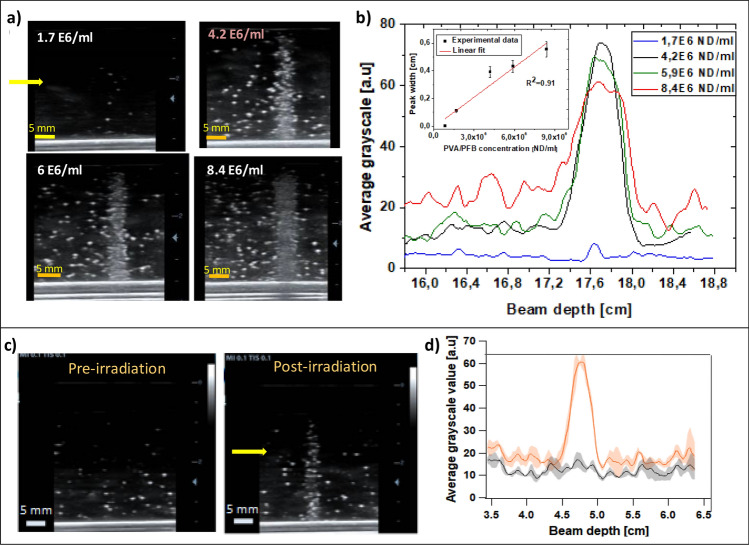
(**a**) US images (@7.5 MHz; MI = 0.1) of phantoms (nanodroplets concentration:1.7 × 10^6^–8.4 × 10^6^ NDs/ml) post 1 Gy C-ions exposure (312 MeV/u, 180 mm); (**b**) Average grayscale value profiles of PVA/PFB NDs phantoms at the different NDs concentrations. The inset plot represents average grey value peak-width (i.e. NDs vaporization signal) as a function of NDs concentration; (**c**) US images of PVA/PFB NDs phantoms pre- and post-irradiation @ 37 °C with C-ion beam range of 50 mm (150.7 MeV/u, 1 Gy, 4 × 10^6^ ND/ml); (**d**) Mean derived vaporization profiles from independent phantoms (orange) compared to the pre-irradiation grayscale signal (black). The shaded areas represent the standard deviation (n = 6).

The NDs concentration is a crucial parameter which determines the lower and upper detection limits within which a given amount of NDs should be high enough to provide sufficient microbubbles density for a measurable acoustic read-out without signal saturation at a given C-ions dose. For this study, the PVA/PFB NDs concentration in the PAM matrix was varied from 8 × 10^5^ to 8. × 10^6^ ND/ml. All the irradiated phantoms underwent the same exposure conditions as detailed in Table 1. Below 4 × 10^6^ ND/ml, which is considered to be the concentration reference, the concentrations seem too low to induce evident vaporization response at the Bragg peak compared to the rest of the phantom after receiving a 1 Gy dose of C-ions. At 4 × 10^6^ ND/ml, the peak in the grayscale profile of the US contrast images at the Bragg peak location is prominent. However, by increasing the NDs concentration even more up to twice the reference value, the generated US contrast at the Bragg peak did not further increase anymore, as the intensity of US signal already reached its maximum. Acoustic shadowing resulted in a diminution of the peak intensity of the derived vaporization profile and in an increase of its broadness (Figs. 4a,b and S5 in the Supplementary Information). We assume that this behavior is mainly engendered by a multiple scattering effect of ultrasound by the bubble cloud around the Bragg peak, causing an overestimation of its broadness. On the other hand, for smaller concentrations of NDs, the Bragg peak profile becomes poorly described.

In a supplementary experiment, the average dose rate was varied by reducing the beam intensity during the exposure of the phantoms with constant NDs concentration to C-ions. Careful analyses of the US contrast data revealed that the NDs’ response was not affected by the dose rate. Fig. S6 in the Supplementary Information indeed show a nearly identical contrast enhancement around the Bragg peak in phantoms exposed to 1 Gy dose with NDs concentration of 4.10^6^ ND/ml at dose rate reduced by half.

### Range verification

Following the evaluation of the C-ions radiation response of the PVA/PFB NDs against several parameters, we investigated the correlation of the observed triggered vaporization with the Bragg peak position. In this regard, we first proceeded to manually displace the phantom container by 1 cm backward within the beam range (i.e. entrance placed at 154 mm instead of 144 mm, see Table 1). As expected, the confined vaporization zone of the NDs is shifted towards the front side of the phantom container and consequently to the left side of the acoustic window in the US images as the transducer’s center is calibrated at the middle of the phantom’s lateral length (Figa. S7a,b and 1c). By comparing the grayscale profiles of the two tested positions (Fig. S7c), we observe a shift in the vaporization fall-off by 1.04 cm, which matches well with the expected distal beam edge. This demonstrates that the phase-transition of the NDs is exclusively induced by the C-ions achieving a maximum LET around the Bragg peak that is above the activation threshold. Moreover, experiment highlights the potential use of the NDs response for range verification with high precision, as a sub-millimeter shift error of 0.4 ± 0.1 mm is obtained which is mainly due to the manual positioning. This result was further confirmed by modifying the beam range from 180 to 50 mm, together with an adaptation of the phantom position (entrance at 20 mm) to ensure that the carbon ions reached the end of their range within the NDs phantom and thus within the imaged acoustic window, as described in Methods section and Fig. 1. The confined activation zone consisting of generated bubbles corresponded to the position of the C-ions Bragg peak and distal dose fall, predicted around 50 mm (Fig. 4). Additionally, the shape of the grayscale vaporization peak was found to be narrower, which is in agreement with physical findings about the relation between the width of the Bragg peak and the beam energy/range^37^. Indeed, it is well known that the Bragg peak gets wider when the nominal energy of the particles beam increases, due to cumulative effects of interactions between incident C-ions and the irradiated material. Accordingly, at low depths, and therefore requiring minor beam energies, the dose profile exhibits a thinner peak.

**Figure 5:**
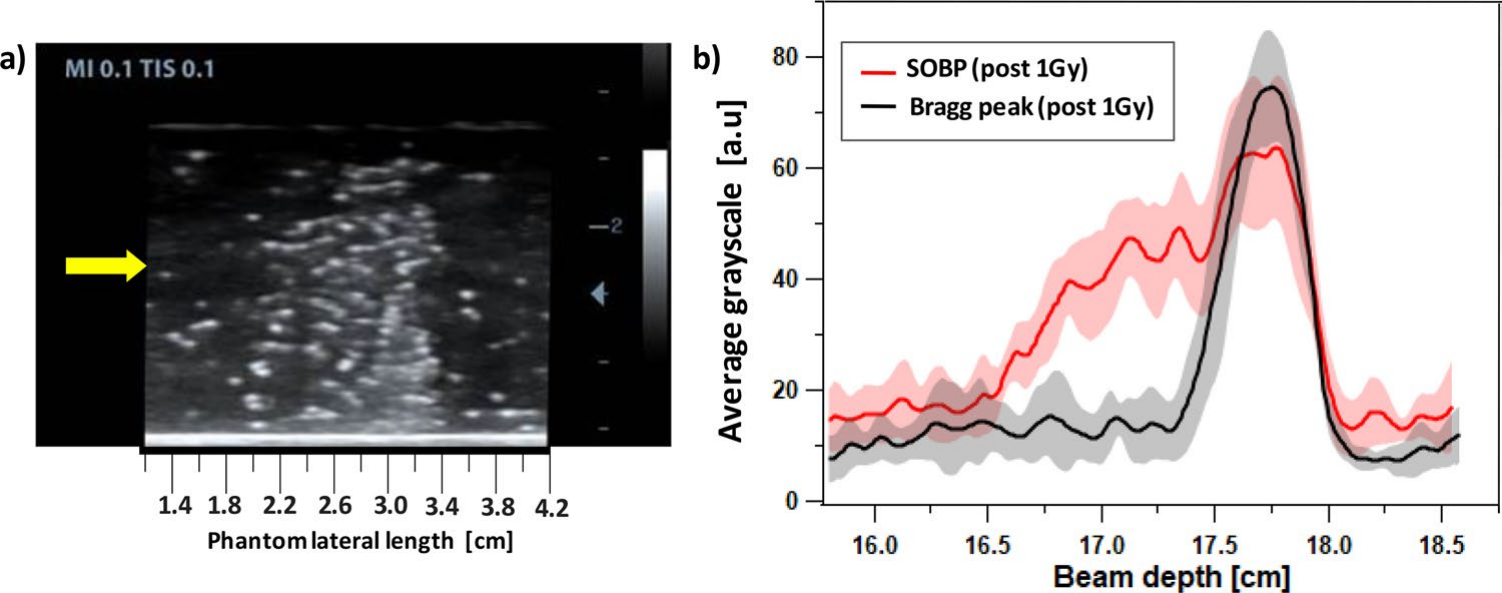
(**a**) US image of SOBP C-ions irradiation of a PVA/PFB PAM phantom @37 °C (1 Gy, 4 × 10^6^ ND/ml, 312 MeV/u, 180 mm); the yellow arrow indicates the beam enteance side. (**b**) Comparison between SOBP and pristine Bragg peak irradiation of NDs post 1 Gy exposure. The shaded areas represent the standard deviations, n = 6).

Finally, we investigated the PVA/PFB NDs behavior in the case of a spread-out Bragg peak irradiation (SOBP) mode, a method usually used in clinics to cover a full tumor volume. In this study, the NDs phantoms received a more uniform and extended C-ions radiation all over the direction of the beam propagation, achieved by stacking multiple Bragg peaks of different depths and weighted energies^38,39^. After being exposed to 1 Gy total dose with cumulative pristine Bragg peaks ranging between 160 and 180 mm, the NDs phantoms exhibited a broad zone of bubbles visible by eye. An example of a depth-resolved US imaging of an SOBP irradiated phantom is illustrated in Fig. 5a. As expected, the image displays a wide zone with enhanced contrast due to the presence of bubbles, whose density is increased towards the largest range of the C-ions exposure. The average profile of the grayscale values after SOBP irradiation (Fig. 5b), can be viewed as the convolution of a broad signal and a peak at the final range. Interestingly, the peak at the end of the vaporization profile overlaps, in terms of shape and width, with the vaporization peak obtained for a single Bragg peak irradiation (180 mm) at the same dose. The lower vaporization contrast-induced intensity observed upon SOBP irradiation was expected as the individual Bragg peaks with lower energies feature a lower number of incident particles^39^.

## Discussion

The above outlined study highlights the specific and high sensitivity of PVA shelled perfluorobutane NDs to C-ions radiation at physiological temperature in tissue-mimicking phantoms. The proof of concept reported herein derives from superheated drop detectors consisting of drops of a metastable liquid halocarbon dispersed in an aqueous/ polymeric matrix. Classically, these detectors can be found in the form of low-cost portable chambers for ionizing radiation detection^40,41^. For example, D’Errico and Di Fulvio reported on neutron detection via an optical readout using vials of superheated viscous emulsions of 100 μm sized C-318 drops^42^. The use of perfluorocarbon phase-change NDs for C-ions dosimetry and range verification offers several advantages that could be relevant for clinical applications in the future. Benefitting from the recent advances of ultrasound contrast agents’ research, these systems were developed mainly as an attractive alternative to conventional microbubbles in order to overcome the bloodstream circulation life^43^. In this regard, NDs could be prospected as non-invasive in vivo dosimeters since they fulfill several criteria: first, the system used for this proof of concept can be transported and easily handled, which is an important factor to limit the cost of the device and of the final radiotherapeutic treatment. The device response to radiation is instantaneous and the US readout is direct. Second, the composition of NDs can be very similar to already clinically available microbubbles and based on FDA-approved compounds (e.g. PFB is used as the gas core in Optison® and Sonazoid® MBs)^44^. Third, they feature a submicron size compatible with intravenous injection and potentially tumor extravasation. Finally, their liquid core is confined in a biocompatible shell ensuring stabilization and enabling further chemical derivatization for requested theranostic approaches^45–48^. In particular, the aqueous suspensions of PVA/PFB NDs, prepared by pre-condensation of PFB (b.p. − 2 °C) followed by its encapsulation with a crosslinked PVA shell (see Methods) in a sonication-mediated process, have consistently proven to exhibit an exceptional shelf-life and thermal stability even at temperatures highly exceeding 37 °C^26,27,49^. The additional Laplace pressure exerted by the shell’s surface tension keeps the core in its metastable liquid state in the absence of external stimuli necessary to reach the vaporization conditions (e.g. temperature beyond the superheat limit, acoustic pressure, ionizing radiation)^43,50,51^. Besides, the inter-chain polymer crosslinks in the shell prevent NDs from coalescence and contribute to an increase of the elastic-modulus, and therefore their stability^52^. The obtained suspensions exhibit a polydisperse size distribution ranging from 200 to 1000 nm. Spontaneous vaporization events are expected to be more probable for the fraction of droplets with the largest size, since those droplets are only weakly affected by the Laplace pressure. When the liquid → gas transition of the PFB core is triggered acoustically, the radius typically expands by a factor of 10 and the yielded microbubbles demonstrated an acoustic resonance frequency within the operational diagnostic range of ultrasonographic scanners, typically 1–20 MHz, which explains their suitability to provide contrast-enhancement during US imaging. Importantly, the successful design of NDs for ionizing radiation dosimetry is based in the first place on the choice of the perfluorocarbon core. By carefully considering the boiling point and thus the degree of superheat at ambient and physiological conditions, good metastability and sensitivity to the targeted type of ionizing radiation can be achieved. Recently, Falatah et al. investigated similar approach for the feasibility of conventional X-ray radiation dosimetry using condensed perfluorpropane droplets (b.p. − 36 °C) from commercial Definity microbubbles^53^. Although perfluoropropane phase-change contrast agents are expected to be less specific to C-ions due to their low LET threshold which can be reached by secondary particles and electrons, the condensation approach of approved microbubbles could be an easier route to bring this concept closer to clinical applications. The degree of superheat of PFB at physiological temperature, calculated using Eq. (1), is equal to 0.34. The relationship between the operating temperature and the linear energy transfer (LET) threshold to sensitize PFB to various types of charged particles is reported in Heymans et al. The threshold values were predicted from the thermal spike theory as the ratio between the nucleation energy required to generate a critical nucleus within the superheated liquid (W_tot_) and twice the critical radius of the latter (R_c_). Table S3 and Fig. S8 in the Supplementary Information summarize the types of ionizing radiations that could reach LET thresholds required to vaporize superheated PFB at both room and physiological temperatures. Other possible PFC candidates that could be more favorable for lower LET radiations are reported for the sake of comparison, although these would imply more sophisticated production and handling. Noteworthy, these theoretical assumptions may be slightly influenced by the nanodroplets reduced size and shell properties (thickness, shear elastic modulus, viscosity), which confer a higher stability to the system. At 37 °C, the modelled LET required to trigger the vaporization of superheated PFB NDs (i.e. 145 keV/μm) is lower than the actual LET achievable by C-ions when they deposit their maximal dose at the Bragg peak^10,54^. Indeed, depending on the operational beam energy and the traversed medium/tissue, C-ions could generate energy densities up to 880 keV/μm, with a maximal RBE in radiotherapy found between 150 and 200 keV/μm^10^. For these reasons, we expect PVA/PFB NDs to be a sensitive dosimeter and prone to undergo a phase-change triggered by primary C-ions at physiological temperature and clinically-relevant conditions. Our results have indeed shown that PVA/PFB NDs, homogenously dispersed in water-equivalent PAM hydrogel phantoms, were successfully vaporized by C-ions radiation and provided reproducible US contrast read-out around the Bragg peak with a relatively proportional response to the received dose. The linearity range of the NDs response to the C-ions beam, evaluated by offline US imaging (@7.5 MHz), was validated up to a dose of 2 Gy for a NDs concentration of 4 × 10^6^ ND/ml. At higher doses, we hypothesize that the number of NDs undergoing a phase transition keeps increasing. However, proper quantification was hindered by the saturation of the US contrast signal and by multiple scattering of large amount of microbubbles around the Bragg peak. In order to overcome US saturation limitations and extend the linear range to higher doses/concentrations, the US imaging setup could be upgraded into an online high-frame imaging allowing for detecting and tracking the distribution of multiple vaporization events of NDs in real-time during radiation exposure^49^.

**Figure 6:**
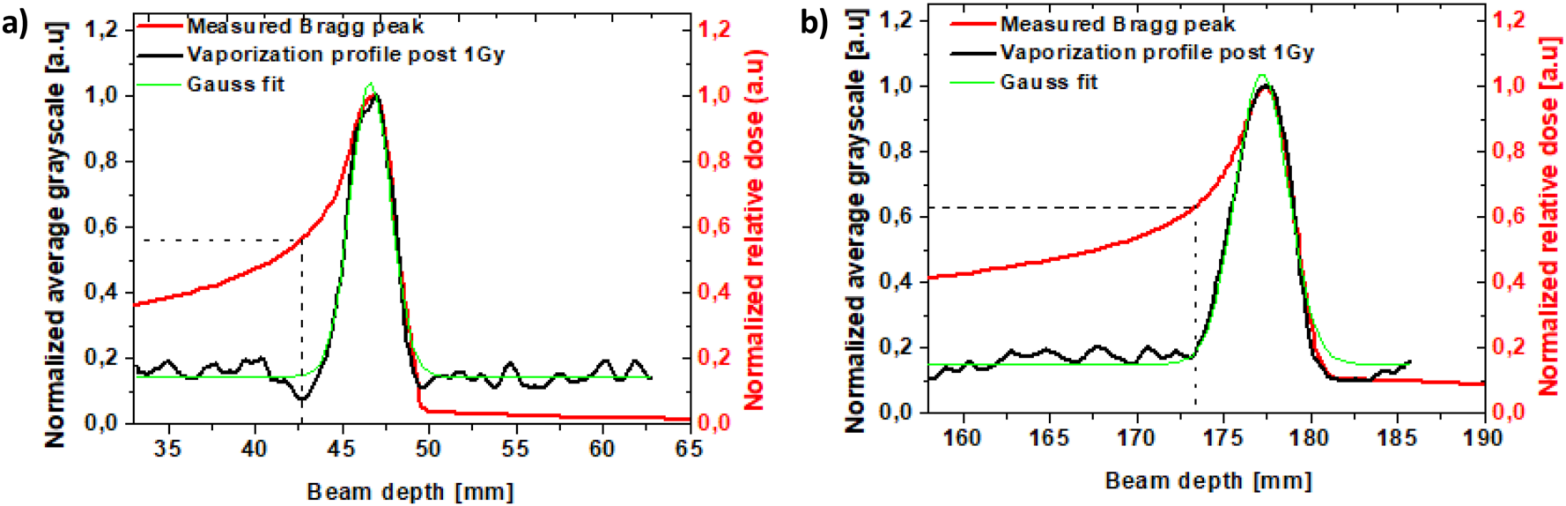
Overlay of the PVA/PFB NDs vaporization profile post 1 Gy C-ions exposure @37 °C and the measured Bragg curve: (**a)** for a beam range of 50 mm and (**b)** for a beam range of 180 mm.

To evaluate the range verification accuracy provided by the confined vaporization zone of NDs around the Bragg peak, we quantified the density of the generated microbubbles along the beam path from the US images post-radiation exposure (1 Gy), and compared the longitudinal depth of both the rise and termination of the vaporization profile to the estimated C-ions stopping distribution at the two tested ranges (i.e. 180 mm and 50 mm). Interestingly, Fig. 6 highlights that the vaporization peaks of PVA/PFB NDs, displayed as grayscale value profiles, coincide perfectly with the measured Bragg peak and with the sharp distal fall-off of C-ions corresponding to each range. The density of the generated microbubbles at the Bragg peaks can be approximated by a Gaussian distribution. It is worth noting that the US contrast reading or equivalently the vaporization of the NDs only starts rising when the Bragg peak reaches about 60% of its maximum (see dashed lines in Fig. 6a,b). This suggests that, at 37 °C, the phase-change of PVA/PFB NDs is only triggered when C-ions deposit their maximal energy. In the plateau region prior to the Bragg peak, the C-ions do not produce sufficient LET to drive the nucleation of the PFB superheated core. In fact, the LET increases as the charged particles slow down and lose their energy^55^. Remarkably, we also did not observe an increase in US generated contrast behind the Bragg peak, despite the potential presence of secondary recoils resulting from nuclear collisions and the presence of a fragmentation tail beyond the Bragg peak. Indeed, the average US grayscale values obtained before and after the NDs’ vaporization peak was similar to the US contrast-noise resulting from small spontaneous vaporization events in the un-irradiated control phantom (see Fig. 3c in the Results section). Therefore, if present at all, the NDs-induced vaporization by secondary particles (mainly protons and neutrons) that usually cause an increase of the dose in the tail part of the Bragg curve is negligible^56^.

The center and R_50_ position (see Fig. S9 in the Supplementary Information) of the vaporization peaks corresponding to the two experimental ranges fit accurately with the absolute position of the Bragg peaks at the maximum and with the 50% drop location yielding a reproducible distal shift smaller than 0.3 ± 0.1 mm, mainly due to the manual positioning errors. In addition, the width of the US grayscale peak after irradiation with 1 Gy dose is in excellent agreement with the Bragg peak broadness quantified by W_80_ (i.e. the width at 80% of the maximum dose level of the C-ions) for both 50 mm and 180 mm beam ranges (see Table 2 and Fig. S9 of the Supplementary Information).

**Table 2:** Analyses of the NDs vaporization profiles (1 Gy, 4 × 10^6^ NDs/ml) vs. the measured Bragg peaks at 50 mm and 180 mm beam ranges.

Range	50 mm		180 mm	
	NDs vap	Measured B.peak in Peak Finder software	NDs vap	Measured B.peak in Peak Finder software
Plateau-to-peak ratio @vap. begin	0.09	0.56	0.17	0.63
x_end_ (mm)	49.7 ± 0.1	50	179.7 ± 0.1	180
x_c_ (mm)	47.3 ± 0.1	46.9	177.2 ± 0.15	177.3
Peak area	121.7	NA	252.5	NA
FWHM (mm)	2.7 ± 0.1	NA	3.6 ± 0.2	NA
Regression coeff. R^2^	0.978	NA	0.976	NA
W_80_ (mm)	2.32 ± 0.1	2.72	2.60 ± 0.2	3.04
Shift @50% drop (mm)	0.2 ± 0.05	NA	0.3 ± 0.1	NA

The results derive from a Gaussian fit function of the grayscale peaks.

While the sharp dose fall-off represents the major advantage of C-ions, it is also a major concern for the organs at risk if the range is not well monitored. The above reported early findings on the highly specific sensitivity of PVA/PFB NDs to C-ions stopping at the Bragg peak confirm their potential for range verification and dosimetry. Moreover, as the perfluorocarbon NDs are well known for their drug loading and controlled release capacities when activated, their phase-transition occurring only at the end of the range could represent an advantageous asset for combining C-ion radiotherapy with controlled and localized drug delivery^57^. As far as we know, range monitors supplying on-line feedback are still missing in C-ions based clinical routine. Computed Tomography is usually adopted as an input for radiotherapy planning and is often performed before the treatment and repeated for verification after a certain number of dose fractions^58,59^. The required safety factors for the treatment typically consider up to 2–3% on the total range because of scan mis-calibration, or morphological changes or uncertainty on patient positioning^14,15^. As an alternative methodology for beam range monitoring, the detection of secondary particles was recently suggested^14,60–62^. Typically, C-ion treatment planning lasts about 4 weeks and consists of total doses up to 60 Gy delivered through several single fractions up to 4–5 Gy each^12^. Recent clinical trials at CNAO tested a developed dose profiler which detects charged secondary fragments that escape the patient to spot morphological changes of tumors after 8 fractions, in patients affected with head neck cancer (Adenoid Cystic Carcinoma)^14^. The presently proposed method uses a combination of NDs and US for beam range verification. Although the use of US imaging suffers from some limitations concerning certain organs such as brain and lungs, it represents a universally available, non-invasive, and cheap diagnosis technique, with capabilities to be used in-line during therapy. In addition, in vivo studies demonstrated that combined ultrasound activated microbubbles with radiation treatments improves the tumor response to the radiotherapy by stimulating vascular disruption^63^.We are aware that this study is an in vitro proof of concept validation adapted to C-ions radiotherapy, but we believe that the outcome is showing large potential as a new ground breaking method.

## Limitation

In the current work we validated the concept of NDs sensitivity to C-ions under physiological and clinical radiation doses without taking size polydispersity into consideration. Future studies will aim in optimizing the NDs, restricting it to the proportion < 500 nm, in order to further gain higher dosimetry spatial precision and favor passive targeting and uptake by tumoral tissues for in vivo purposes. In vivo, where NDs are prone to the blood shear-stress and diffusion, their accumulation in tumoral sites of interest and surrounding healthy tissues will require further deep investigations. Indeed, the nanodroplets’ inohomogeneous distributions in real tissue could affect the accuracy of localizing and quantifying the radiation-triggered vaporization by US imaging.

## Conclusion

We demonstrated in this contribution the feasibility of an ultrasound-assisted procedure for C-ions dosimetry and range verification by means of injectable superheated phase-change contrast agents through in vitro experiments. The ultrasound-contrast generation from PVA shelled perfluorobutane nanodroplets after vaporization upon C-ions irradiation was evaluated in soft tissue-mimicking phantoms at physiological temperature (i.e. 37 °C) and with clinically relevant C-ions doses up to 4 Gy. In contrast to our previous proof-of-concept studies in proton beams, where the proton range was detected indirectly (i.e. through visualization of nanodroplet vaporization by secondary recoils), herein, the nanodroplets showed an extremely promising radiation sensitivity, selective to the Bragg peak, in both pristine and spread-out Bragg peak irradiation modes. Exposing the phantoms to C-ions led to a strong contrast increase with high precision at the Bragg peak location, attributed to nanodroplets conversion into microbubbles. The offline ultrasound-based vaporization detection critically depends on the radiation dose and on the concentration of nanodroplets in the phantom, while the vaporization response was found to be unaffected by the dose rate. The quantification of the grayscale generated contrast (integrated profile) was linearly related to the C-ions dose below 2 Gy, for a nanodroplets concentration of 4 × 10^6^ ND/ml. Overall, the results prove the potential of PVA perfluorobutane nanodroplets for C-ions dosimetry and range verification. Future work aims at confirming these early findings in preclinical experiments and at implementing a transition to online ultrasound imaging to better quantify the radiation response over a broader dose and concentration range.

## Supplementary Information

Supplementary Information.

## Data Availability

All data generated or analyzed during this study are included in this published article (and its Supplementary Information files).
