# Supplementary material for: Ultrasound-assisted carbon ion dosimetry and range measurement using injectable polymer-shelled phase-change nanodroplets: in vitro study
Source: Sci Rep. 2022 May 14;12:8012. doi: 10.1038/s41598-022-11524-x (PMC9107472; doi:10.1038/s41598-022-11524-x)
Supplement: Supplementary file 1 — Supplementary Information. [file 41598_2022_11524_MOESM1_ESM.pdf]

Supplementary Information

Ultrasound-Assisted Carbon Ion Dosimetry and Range Measurement Using Injectable Polymer-Shelled Phase-Change Nanodroplets: In Vitro Study

Yosra Toumia[1,2*], Marco Pullia[3], Fabio Domenici[1,2], Angelica Facoetti[3], Michele Ferrarini[3], Sophie V. Heymans[4,5,6], Bram Carlier[7], Koen Van Den Abeele[4], Edmond Sterpin[7], Jan D'hooge[5], Emiliano d'Agostino[8], and Gaio Paradossi[1,2]

[1]Department of Chemical Science and Technologies, University of Rome Tor Vergata, Rome 00133, Italy
[2] National Institute for Nuclear Physics, INFN sez. Roma Tor Vergata, Rome 00133, Italy
[3] Fondazione CNAO, The National Center of Oncological Hadrontherapy, Pavia 27100, Italy
[4]Department of Physics, KU Leuven Campus Kulak, Kortrijk, Belgium
[5]Department of Cardiovascular Sciences, KU Leuven, Leuven, Belgium
[6]Biomedical Engineering, Department of Cardiology, Erasmus MC University Medical Center, Rotterdam, The Netherlands
[7]Department of Oncology, KU Leuven, Leuven, Belgium
[8]DoseVue, Hasselt, Belgium

**Corresponding Author: Yosra.Toumia@uniroma2.it*

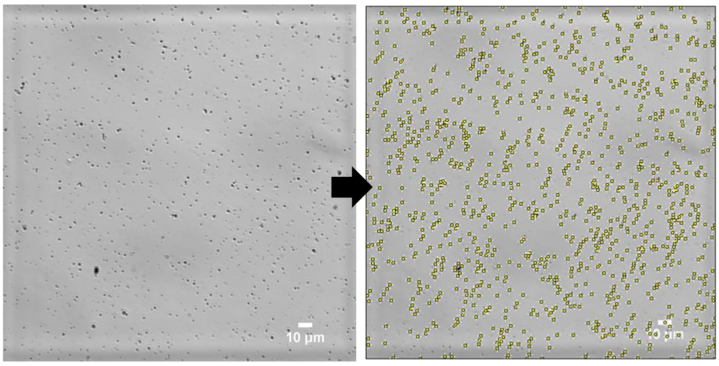

Figure S1. Optical microscopy image of PVA/PFB NDs in a Neubaur chamber (objective 40×) used for automatic count estimation using Image J freeware.

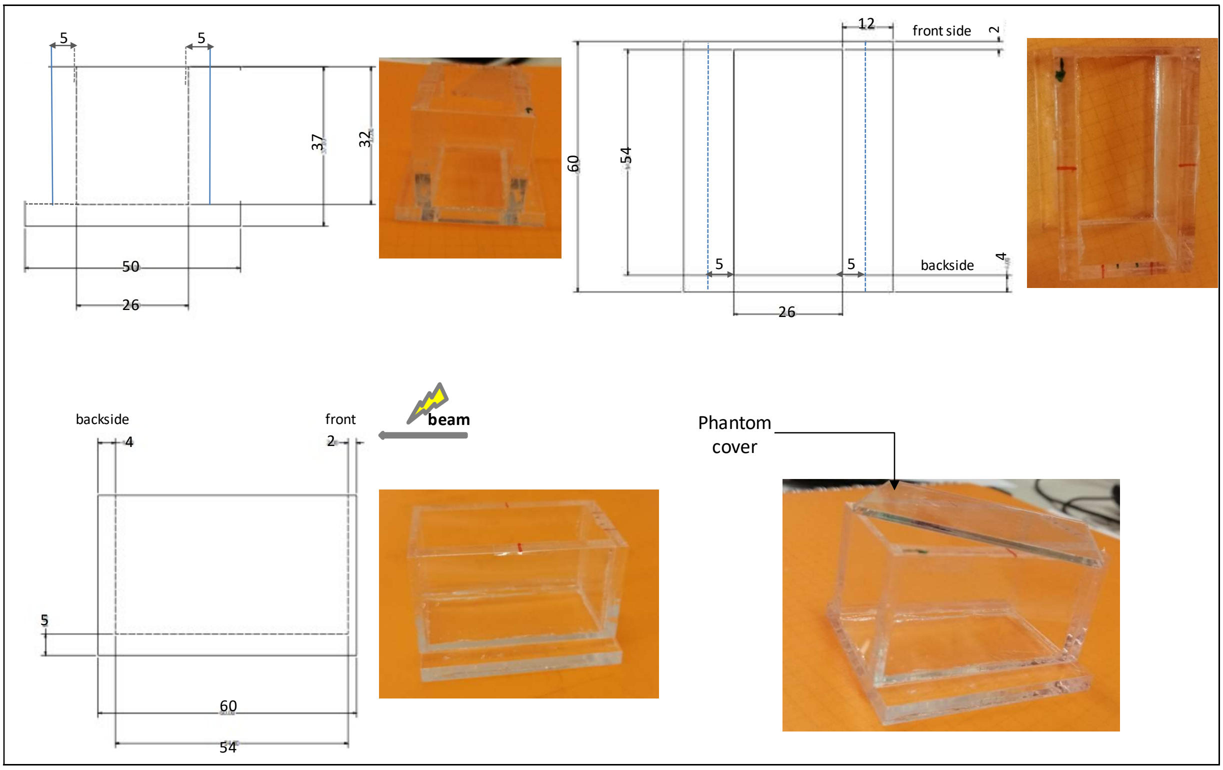

Figure S2. Full description of the PMMA phantom containers (the given unit of measurement is in mm). The phantom's lateral length in the manuscript refers to the internal length of PMMA container. Front and backside refer to the thinner (2 mm) and thicker (4 mm) wall, respectively.

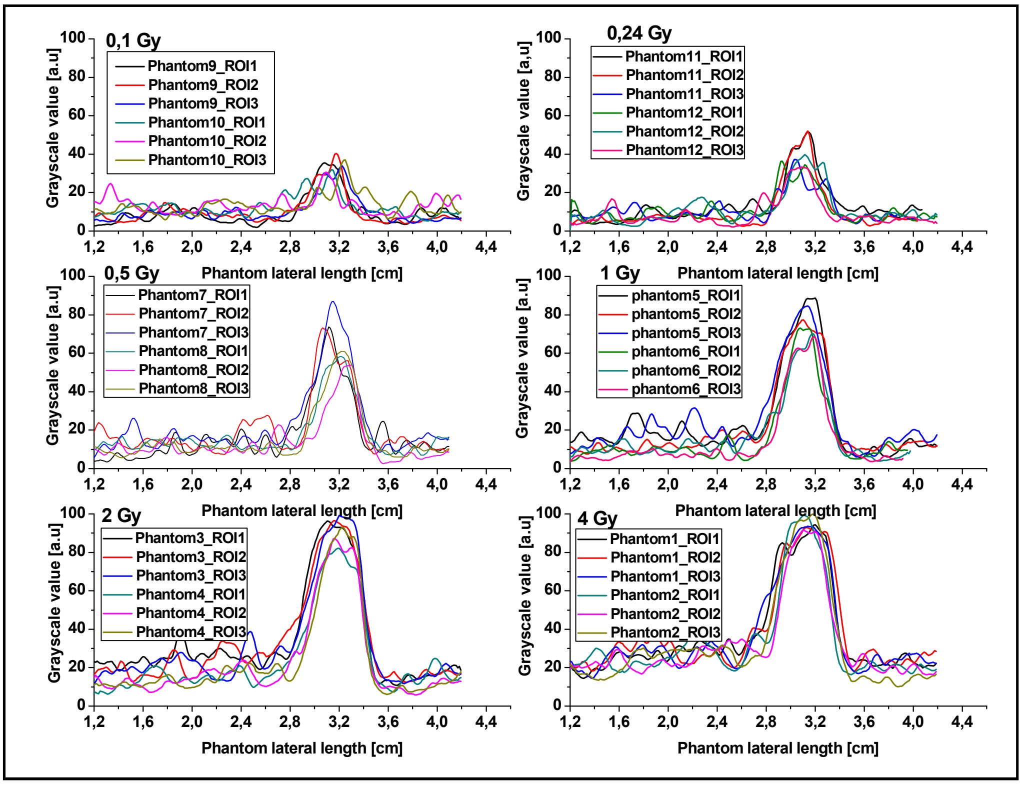

Figure S3. Extracted grey value profiles from the scanning of two phantoms containing PVA/PFB NDs post exposure @37°C to individual C-ions doses ranging between 0.1-4Gy at the Bragg peak (beam range=180mm).

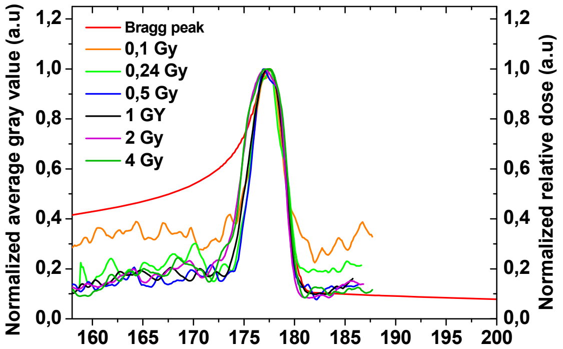

Figure S4. Comparison of measured C-ions Bragg peak and grayscale profiles derived from PVA/PFB triggered vaporization at different C-ions doses between 0.1-4Gy (@37°C, $4{\cdot}10^6$ ND/ml, 312 MeV/u).

The dose-response equation is: $A1 + \frac{A2-A1}{1+10^{((LOGx_0-x)\cdot p)}}$ (Equation S1)

where A1 and A2 are the lower and upper asymptotes, respectively, x is the dose expressed in Gy, $LOGx_0$ corresponds to the median dose value in the curve, and p is the hill slope.

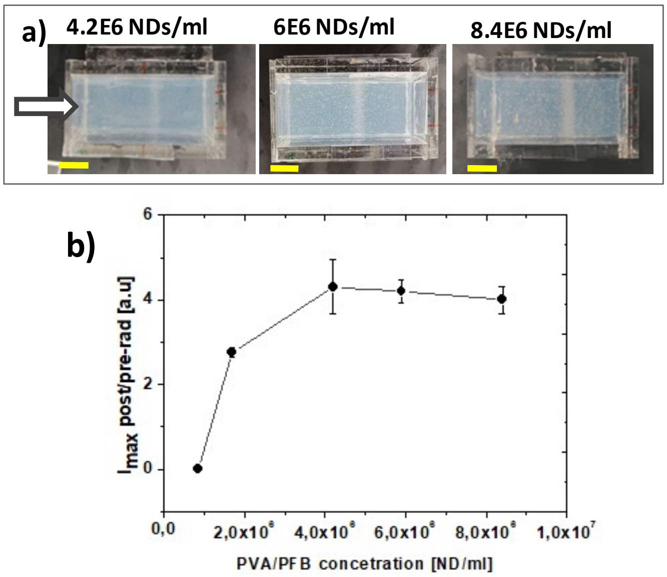

Figure S5. a) Photographs of three PVA/PFB NDs phantoms with varied concentrations of nanodroplets post-irradiation @37°C with 1 Gy C-ions dose at the Bragg peak (beam range=180mm, 312 MeV/u, the arrow indicates the beam entrance); the yellow scale bars correspond to 10 mm. b) post/pre-radiation contrast enhancement at the peak ROI as function of NDs concentration (the line is a guide to the eye). The error bars correspond to the standard deviation of the US images taken from two identical phantoms of NDs (n=6). A quantification of the ratio between grayscale value intensity pre-and post-radiation was adopted to account for the differences in the noise background resulting from spontaneous vaporization, since the higher the concentration of NDs dispersed in the PAM matrix the higher is the obtained background signal.

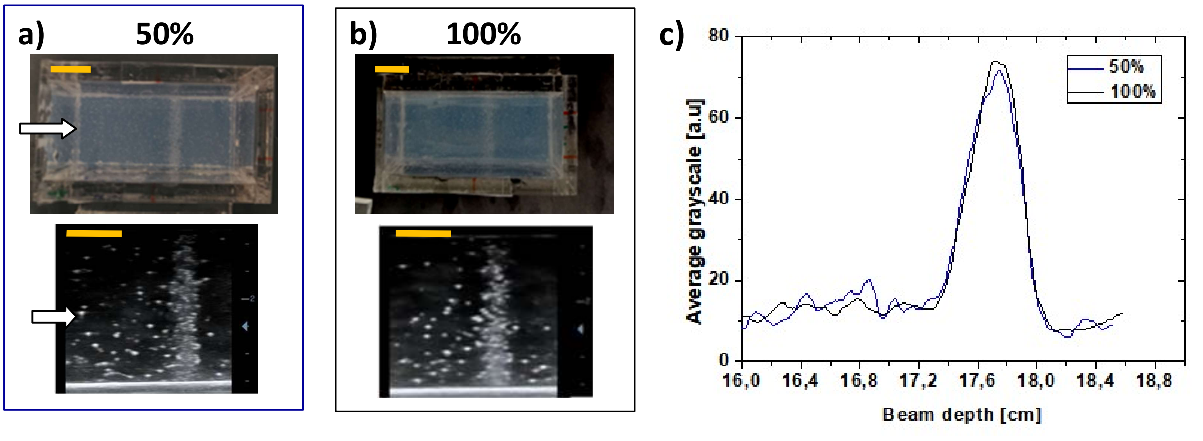

Figure S6. Illustration of the independency of PVA/PFB NDs (concentration ≈ $4{\cdot}10^6$ NDs/ml) to the average dose rate of C-ions: a) and b) photograph and US imaging of NDs phantom post 1Gy exposure (beam range 180 mm, the arrows indicate the beam entrance side) at relative dose rates of 50% and 100%, respectively (the yellow scale bars correspond to 10 mm); c) corresponding average grayscale value profiles obtained for the two dose rates The 50% average dose rate corresponds to a reduced beam intensity reduced by a factor of 2.

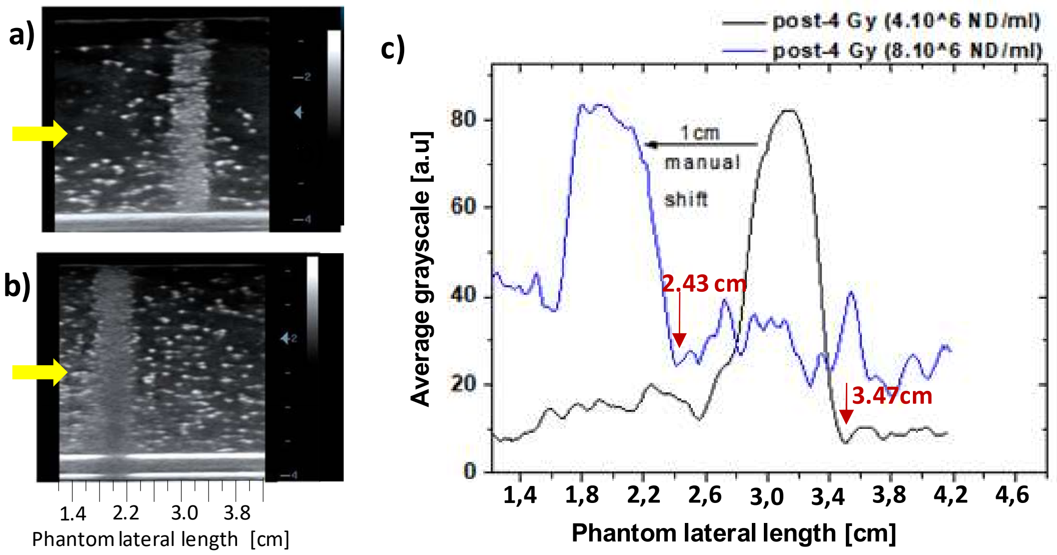

Figure S7. Ultrasound images (acquired @7.5 MHz and MI=0.1) of PVA/PFB NDs dispersed in PAM phantom post-4 Gy irradiation with C-ions (@37°C, 312 MeV/u, 180mm range): a) phantom container positioned at 144 mm of the beam depth ($4{\cdot}10^6$ NDs/ml); b) phantom container shifted manually by 1cm (position 154 mm of the beam depth, $8{\cdot}10^6$ NDs/ml); c) average grey value profiles of the phantoms positioned at 154mm (blue) and at 144mm (black) of the beam depth. Abscissas refer to the lateral length of the phantom with respect to the probed acoustic window. The red arrows indicate the absolute positions of NDs' vaporization fall-off in the phantom. The dissimilarity in intensity between the two grayscale profiles is due to the difference in NDs' concentration.

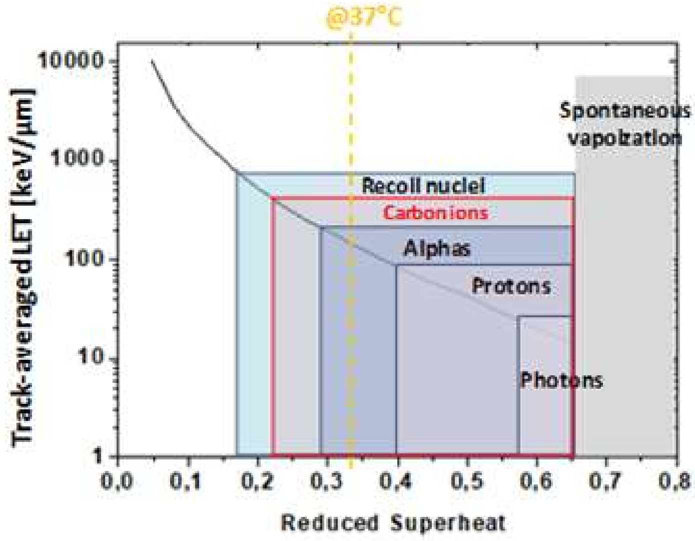

Figure S8. Linear energy transfer (LET) threshold, obtained from the thermal spike theory as the ratio between the nucleation energy (W_{tot}) and twice the critical radius (R_c), for superheated perfluorobutane as a function of the degree of superheat. Rectangular selections highlight the limit zones where droplets are sensitized to specific ionizing particles.

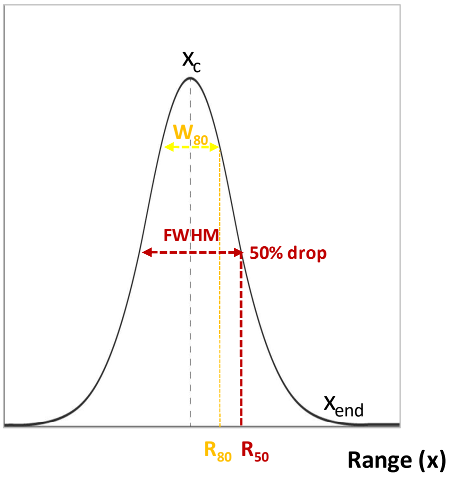

Figure S9. Definition of the Gaussian fit output parameters of the vaporization profiles of PVA/PFB NDs for Bragg peak curve comparison.

Table S1. Average grayscale peak integrals at various C-ions doses (180 mm range) from 0.1 to 4 Gy (without fit).

Dose (Gy)	Area (a.u)	Width (cm)	X_{center} (cm)	Height (a.u)	X_{end} (cm)	$X_{beginning}$ (cm)
**0.1**	8.14709	0.31679	17.76383	21.46224	18.21679	17.25475
**0.24**	10.64053	0.36109	17.76311	30.09379	18.09038	17.351
**0.5**	16.87285	0.34628	17.69884	48.15336	18.07451	17.39581
**1**	25.34641	0.39011	17.75171	64.41606	18.16382	17.26687
**2**	34.02848	0.44118	17.71153	75.6267	18.07518	17.15397
**4**	34.82845	0.46499	17.73959	72.96385	18.12746	17.15778

Table S2. Peak analyses using Gaussian fit from grayscale profiles at different doses.

	Dose (Gy)	Peak area (a.u)	Peak center x_c (cm)	Peak height y_c (a.u)	FWHM (cm)	R^2 gauss fit
**180 mm**	**0.1**	6.621	17.727	19.782	0.3144	0.962
	**0.24**	10.738	17.727	30.381	0.3320	0.975
	**0.5**	18.255	17.729	52.005	0.3298	0.977
	**1**	25.251	17.719	66.252	0.3581	0.976
	**2**	33.756	17.686	79.442	0.3.992	0.956
	**4**	33.234	17.697	75.415	0.4140	0.957
**50mm**	**1**	12.004	4.734	41.656	0.2715	0.977

Table S3. Required LET threshold values for superheated perfluorocarbons at room temperature and 37°C according to the thermal spike theory. HFP and PFP denote 2H-heptafluoropropane and perfluoropropane, respectively.

	PFB (b.p=-2°C)		HFP (b.p=-15°C)		PFP (b.p=-37°C)
**Temperature**	**25°C**	**37°C**	**25°C**	**37°C**	**25°C**
**Superheat (s)**	0.23	0.34	0.35	0.45	0.57
**LET threshold (keV/µm)**	370	145	124	57	25
**Ionizing radiations**	Heavy ions (carbon, oxygen)	Heavy ions, α particles	Heavy ions, α particles	Protons, He, Heavy ions, α particles	Electrons, protons, He, Heavy ions, α particles
